# Facebook, Instagram, Pinterest and co.: body image and social media

**DOI:** 10.1186/2050-2974-3-S1-O22

**Published:** 2015-11-23

**Authors:** Genevieve Pepin, Natalie Endresz

**Affiliations:** 1 Deakin University, Australia

## 

Evidence linked exposure to internet appearance-related sites to weight dissatisfaction, drive for thinness, increased internalisation of thin ideals, and body surveillance with Facebook users having significantly higher scores on body image concern measures (Tiggemann & Miller, 2010, Tiggemann & Slater, 2013). This study explored the impacts of social media on the body image of young adults aged 18-25 years. A total of 300 students from a Victorian university completed a survey including questions about the use of social media and 2 measures of body image: The Objectified Body Consciousness and both female and male version of the Sociocultural Attitudes towards Appearance Questionnaire 3. Results showed participants mostly used Facebook to keep in touch with friends and family. While using social media, they felt pressure to lose weight, look more attractive or muscular, and to change their appearance. Correlations were found between Instagram and concerns with body image and body surveillance, between Pinterest and body shame and appearance control beliefs and between Facebook and Pinterest and perceived pressure. Findings contribute to the growing body of knowledge about the influence of social media on body image and new information for the development of social media literacy programs addressing negative body image.

